# The approach to developing Ireland’s first national health protection strategy and lessons learnt, December 2021 to October 2022

**DOI:** 10.2807/1560-7917.ES.2024.29.14.2300326

**Published:** 2024-04-04

**Authors:** Ciara Kelly, Joan Gallagher, Lola Odewumi, Robert Conway, Mary E Black, Kristin Concannon, Louise Marron, Lorraine Doherty

**Affiliations:** 1 PricewaterhouseCoopers Ireland, Dublin, Ireland; 2 Health Service Executive-Health Protection Surveillance Centre, Dublin, Ireland; 3 National Health Protection, Health Service Executive-Health Protection Surveillance Centre, Dublin, Ireland; 4 School of Medicine, University of St. Andrews, St Andrews, Scotland, United Kingdom; 5 Strategic Public Health, Office of Chief Clinical Officer, Health Service Executive, Dublin, Ireland

**Keywords:** Ireland, Public Health Policy

## Abstract

The COVID-19 pandemic highlighted the importance of strengthening health protection worldwide. To address this as a public health priority in Ireland, between December 2021 and October 2022 the first national Health Protection Strategy (2022–2027) for the Irish Health Service Executive (HSE) was developed. We describe the approach taken to develop a first national health protection strategy for Ireland, and highlight the key lessons learned. Key steps in strategy formation included detailed stakeholder analysis, exploration of the context for the strategy and development of a comprehensive consultation plan. Two stakeholder consultation workshops were held. The first focused on defining strategic vision, aim and objectives, the second verified objectives and identified enablers. A subsequent e-consultation invited feedback from wider stakeholders. The published strategy outlines 10 strategic objectives and 11 enablers. Key lessons identified from the strategy development process include the importance of clear leadership and oversight, the value of identifying the context for change, ensuring adequate consultation planning, taking a multidisciplinary approach with strong stakeholder engagement and the need to maintain a strategic perspective. Lessons from our experience can support colleagues internationally to strategically set out their priorities for health protection beyond COVID-19.

## Background

Health protection is a core domain of public health medicine in Ireland. Recognised as an essential public health function by the World Health Organization (WHO), health protection includes the prevention and control of infectious disease, environmental and radiation risks and the emergency response to major incidents and health threats [1,2]. The COVID-19 pandemic highlighted the global threat to population health posed by infectious agents. In Ireland, it particularly highlighted the critical importance of a national public health service with the capability to provide a robust, resilient and dynamic health protection response to all hazard health threats [3]. ‘All hazards’ is defined by the WHO as a concept which recognises that although hazards can vary in origin, they often challenge health systems in similar ways [4].

In Ireland, the national Health Service Executive (HSE) delivers publicly-funded health services [5]. The Public Health workforce, as part of the HSE, provides public health expertise and services at local and national level [6]. The health protection service within the HSE is further described in [1]. The COVID-19 pandemic resulted in a significant expansion of the Public Health workforce in Ireland, which occurred in the context of an ongoing programme of reform for this workforce. This included the establishment of the role and Office of the National Clinical Director for Health Protection (NCDHP). To guide future priorities for this workforce within health protection, the HSE Office of the Chief Clinical Officer (CCO) required the development of a National Health Protection Strategy for Ireland 2022–2027. This strategy was the first of its kind in Ireland, with a stated aim of addressing threats to population health from all hazards, not solely those posed by infectious diseases [1]. The strategy outlines key objectives and priority actions for the HSE Public Health workforce to take, in collaboration with key stakeholders, to achieve the protection of the population of Ireland from all health protection hazards [1].

Here we describe the approach taken to develop the first national health protection strategy for Ireland and highlight the key lessons learned.

### Strategy development approach

**Figure 1:**
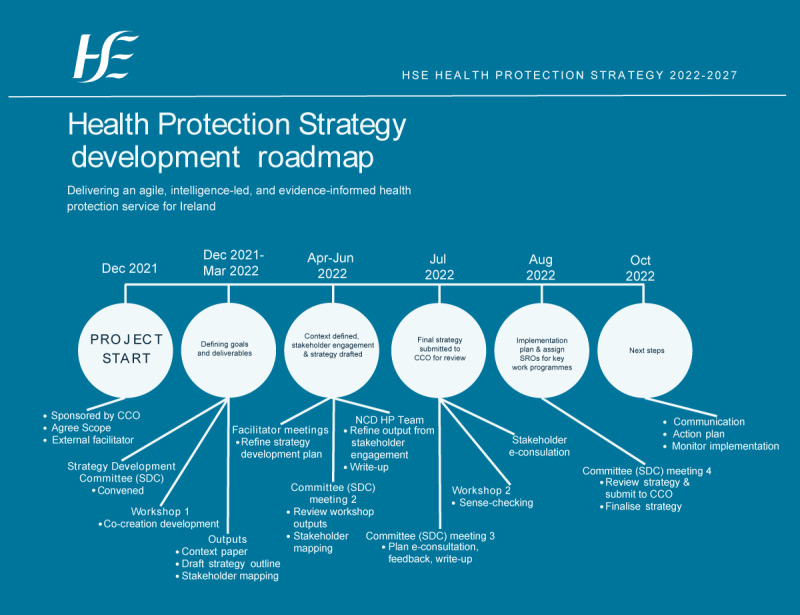
Roadmap for the development of the national Health Service Executive Health Protection Strategy 2022–2027, Ireland, December 2021–October 2022

A mandate was issued by the HSE CCO in December 2021 to the then NCDHP, for the development of a HSE health protection strategy. The HSE CCO was the sponsor (i.e. owner) for this project. Figure 1 presents a summary road map of the 11-month strategy development process.

A multidisciplinary Strategy Development Steering Committee (SDSC) was convened by the NCDHP in January 2022 to oversee and provide expert input to strategy development. The list of members is available in the published strategy [1]. The terms of reference for the SDSC are available in [7]. The SDSC met throughout the process of strategy development, between January and August 2022. Its work was supported by a project working group (PWG), led by the NCDHP.

Broadly, our strategy development approach was defined by addressing four key questions, underpinned by extensive stakeholder consultation and collaboration (Box).

Box **The four key questions used to define our strategy development approach**

- Where are we now? (identifying the context for change).
- Where do we aim to be in five years’ time? (setting the strategic aim and vision).
- How are we going to get there? (collaboratively agreeing strategic objectives and enablers).
- How will we know when we have got there? (identifying performance measures for objectives).

The NCDHP and PWG developed a context paper which explored the health protection and wider public health context, nationally and internationally, for the proposed health protection strategy (first draft March 2022 [7]). Key points addressed in the context paper included the statutory functions of health protection in Ireland, the Public Health Reform Programme ongoing in Ireland at the time, key learning from the COVID-19 pandemic response, relevant national policy publications and the international context focusing on strategic health protection or broader public health priorities identified by other countries informed by a rapid literature review [7].

### Stakeholder consultation process

Stakeholder identification, mapping, analysis and engagement planning was guided by the HSE Health Services Change Guide [8], and periodically reviewed throughout the consultation process. An experienced external facilitator was engaged to advise on the consultation process and facilitate consultation workshops. The consultation process included two in-person consultation workshops (March and June 2022), and one online (e-)consultation (19 July to 5 August 2022) using the Qualtrics survey platform [9]

In advance of each workshop, the PWG and external facilitator agreed on the aims, objectives, expectations, required inputs (e.g. prereading), materials, desired outputs and logistics. Workshop 1 aimed to invite and explore participant input and discussion on key areas relevant to strategy development, largely through facilitated group-based discussion of key questions (available as Supplementary material), followed by a plenary session on health protection enablers. Workshop 2 aimed to seek views from participants on the draft objectives and enablers, also through facilitated group-based discussion. Two detailed reports with thematic analysis of outputs from each workshop were produced, which informed strategy development.

The e-consultation sought feedback on the draft strategy from a broader range of stakeholders beyond those engaged for workshops, e.g. across HSE Public Health, other HSE divisions, government agencies and other external agencies. The profile of all stakeholders consulted throughout the strategy development process is available in the published strategy and included stakeholders internal and external to the HSE [1].

Overall, there was strong engagement with the strategy development process from the multidisciplinary SDSC and wider stakeholders. Strategy development consultation workshops were well attended, with 23 and 29 participants at the first and second workshops, respectively (excluding the PWG members). The opportunity to collaborate in-person and discuss potential strategic priorities in groups was consistently noted across participant feedback as the most useful aspect of the first workshop.

### Selecting strategic objectives and enablers

**Figure 2:**
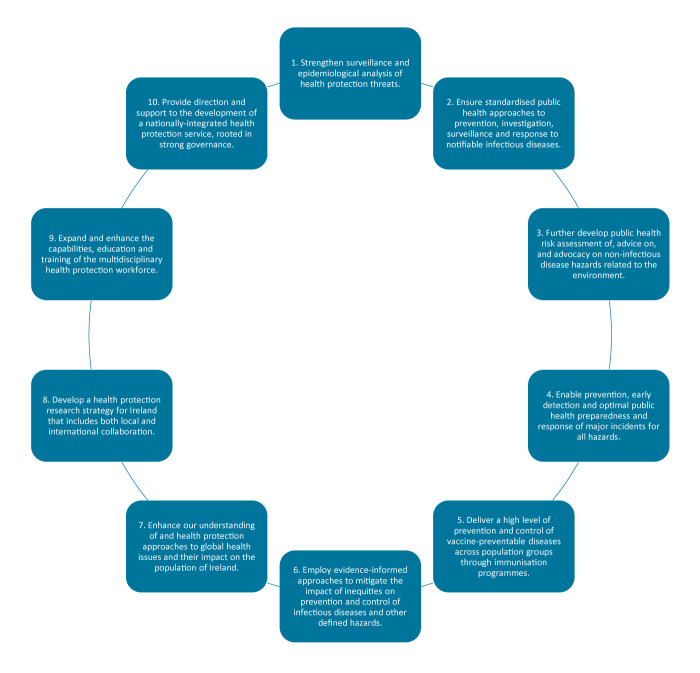
The Health Service Executive Health Protection Strategy 2022–2027’s 10 strategic objectives, Ireland, December 2021–October 2022

**Figure 3:**
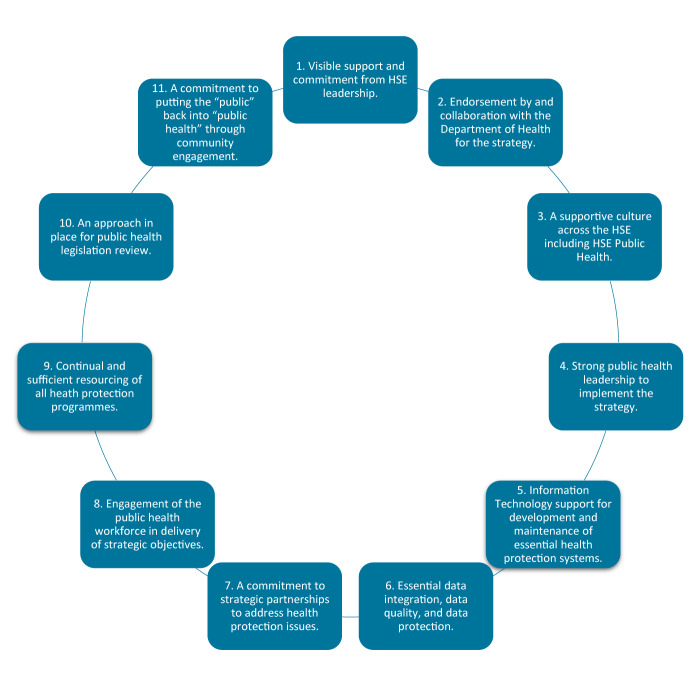
The 11 key enablers of the Health Service Executive Health Protection Strategy 2022–2027 that were identified in order to achieve the strategy’s objectives, Ireland, December 2021–October 2022

Driven by the PWG and SDSC, the workshop consultation process resulted in a distillation of input from stakeholders over a period of 6 months into 10 strategic objectives and 11 key enablers. (Figures 2 and 3) Selection of strategic objectives was based on a combination of evidence and collaborative stakeholder discussion. Relevant contextual factors identified in the context paper were reviewed by participants of the first workshop. These factors informed an original list of proposed objectives. Strategic priorities identified in international publications, as part of the context paper, were also considered. Consultation on this draft list, and proposed enablers (as identified in the first workshop), was a key focus of the second workshop, with key aspects discussed and debated by attendees. This resulted in an evidence-informed, collaboratively chosen, list of 10 strategic objectives which prioritised core areas for health protection to deliver the greatest impact for the population, underpinned by key enablers. Objectives were not graded in terms of importance, nor were any considered controversial. Overall, the objectives were collectively deemed relevant to achieve the stated vision of the strategy, to protect the health and wellbeing of people in Ireland against all health protection hazards.

HSE: Health Service Executive.

The key enablers are described in full in [1].

There were 71 valid responses to the e-consultation survey, of which 38.0% (n = 27) were from HSE Public Health, 32.4% (n = 23) from another HSE division, 18.3% (n = 13) from a government agency and 11.3% (n = 8) from another external agency. Seven additional feedback responses were received by email. There was broad agreement in the e-consultation feedback with the content of the strategy.

Following finalisation of the strategy by the PWG, review and approval by the SDSC and NCDHP, the document was circulated to the project sponsor (HSE CCO) and the Department of Health in Ireland for approval and endorsement. The strategy was formally launched in October 2022 [10].

### Considering the international context

Our key lessons can be applied to, and inform, future similar strategic projects in Ireland and internationally. A notable finding from the review of international grey literature on health protection and broader public health strategy was that while there are many published strategies across public health and health protection, there was a relative absence of detail on the strategy development process itself. This marks a gap in the literature, which our study contributes towards filling. This gap also makes comparing and contrasting our approach challenging.

We can, however, consider how our strategic objectives align with those set out by others. The rapid review of grey literature undertaken as part of our context paper focused on health protection literature from high-income countries, Organisation of Economic Co-operation and Development countries and the WHO European Region. Relevant publications were identified from Australia [11-16] England [17-19], France [20,21], Germany [22], New Zealand [23], Qatar [24], Scotland [25], Wales [26-28], Northern Ireland [29,30], the United States [31-33], the European Centre for Disease Prevention and Control [34] and the WHO [35]. These were predominantly published before the COVID-19 pandemic, and in the majority, did not state an explicit all hazards perspective on health protection [7]. This is in contrast to our strategy which takes an all hazards approach to protecting population health, in the context of an ongoing pandemic response and recovery. There were, however, common themes and alignment between many of our strategic priorities and those identified by others internationally – in particular, infectious diseases, immunisation, health inequities, surveillance, partnerships, environmental hazards, workforce, partnerships, strengthening capacity and research [7].

### Key lessons learnt

Strong, consistent leadership by the NCDHP on strategy development, clear governance for strategy delivery and multidisciplinary SDSC oversight were crucial to the success of this project. The issuing of a mandate by the HSE CCO supported the NCDHP in convening and leading the SDSC, resourcing the development process and emphasised the necessity and timeliness of the activities required to develop the strategy. This was also important given a changing political and operational context over the course of strategy development. For example, there were changes in Department of Health leadership during this time, and a new National Director of Health Protection appointed just before the official launch of the strategy [36-38]. This dynamic context and potential associated impacts were consistently acknowledged with stakeholders.

A foundational first step in the development of the strategy was to identify and explore the national and international context for strategy development. This was achieved through consultation with SDSC stakeholders on relevant context factors at national level, and a review of international grey literature. The rapid review of grey literature identified several relevant strategic and policy documents for health protection and public health more broadly. This document facilitated a comprehensive understanding of international strategies and national contextual factors that informed the development of national strategic priorities.

Prioritising preparation of a detailed consultation plan was crucial to its successful implementation and the breadth of stakeholder input achieved. The experience and guidance of the appointed external facilitator was invaluable in both this planning process, and in the workshops. The presence of the external facilitator also had a positive, energising effect on workshop participants.

### A multidisciplinary approach with strong stakeholder engagement

The multidisciplinary nature of the SDSC and strategy consultation process was central to the production of a comprehensive, well-structured and priority-focused strategy, and resulted in the provision of a broad range of relevant views on health protection in Ireland. The inclusive stakeholder identification and analysis undertaken throughout the strategy development process was key to achieving this multidisciplinary input and oversight from the SDSC and successful engagement with the wider national public health community. The degree of engagement and enthusiasm from stakeholders, internal and external to Public Health, was notable throughout all aspects of the consultation process. The opportunity to have such a high level of positive engagement was invaluable, particularly in relation to identification of and shaping strategic health protection priorities. The strategy development process began 2 years into a sustained COVID-19 pandemic response, and despite this, stakeholders involved in the development of the strategy continued to express enthusiasm for a shift in the prevailing health protection perspective, towards creating a proactive future-focused framework for service delivery. This highlights the value in engaging stakeholders early and comprehensively in strategy development.

High levels of expectation and energy from stakeholders brought the need to manage expectations and maintain a high-level strategic view throughout the process of strategy development. This was largely achieved through planning in advance for all stakeholder consultations, and provision of a consistent message regarding the scope of the strategy itself. For example, in the consultation workshops, a ‘parking lot’ space was created where items raised that were considered not within scope of the strategy were captured by documenting on a flip chart and later noted for the attention of the NCDHP. This was particularly helpful for managing discussions that strayed into implementation and operational delivery of strategic objectives.

### Looking to the future – strategy implementation

Led by the Director of National Health Protection, the objectives of the HSE National Health Protection Strategy will be delivered over the 5-year period 2022–2027. Actions defined for each strategic objective are used to inform close monitoring of strategy implementation progress and allocation of resources. The first annual report on implementation progress was published in October 2023, in conjunction with the first HSE Public Health Health Protection Conference, with work now underway for year 2 [39]. As regards funding and legislation, implementation of the strategic objectives will be mainly funded from within existing resources. Where additional resourcing is required, funding will be sought as part of national service planning mechanisms. The Medical Officer of Health legislation underpins the work required to protect populations and sets out the obligations of the statutory health protection service in Ireland [1,40].

## Conclusion

There is much to be learned from the process of strategy development – specifically, what worked well, what did not and what can be improved upon for the future. This learning is relevant not only in Ireland, but also within the wider European and indeed international public health community. As the world continues adapt to the consequences of the COVID-19 pandemic and prepares to face future threats, it is imperative that public health organisations and governments take the time to strategically consider and set out priorities for health protection and public health action beyond COVID-19, informed by learnings from the pandemic. The lessons learned from the experience of this health protection strategy development process in Ireland can inform strategy development in other countries, and ultimately, benefit the protection of populations from all public health hazards.
